# Finite element analysis in dentistry: a comprehensive narrative review of biomechanical principles, clinical applications, and future perspectives

**DOI:** 10.3389/fdmed.2026.1902992

**Published:** 2026-07-30

**Authors:** Niranjan Harikrishna, Avishikta Banerjee, Shreya Chandrashekhar, Nishmitha N. Hegde, Chaithra Lakshmi V, Mithra N. Hegde

**Affiliations:** 1 Nitte (Deemed to be University), AB Shetty Memorial Institute of Dental Sciences (ABSMIDS), Department of Conservative Dentistry and Endodontics, Mangalore, India; 2 AB Shetty Memorial Institute of Dental Sciences, Nitte (Deemed to be University), Mangalore, India

**Keywords:** dental biomechanics, endodontics, finite element analysis, implantology, orthodontics, patient-specific modelling, prosthodontics, stress distribution

## Abstract

Finite Element Analysis (FEA) is a well-established numerical modelling technique increasingly adopted in dental research to model the biomechanical behaviour of oral structures under functional and parafunctional loading conditions. This narrative review aims to (1) trace the historical development of FEA from its engineering origins to dental applications, (2) describe the methodological framework of FEA with emphasis on dental-specific modelling considerations, (3) comprehensively survey current applications across all major dental specialties, and (4) critically appraise the technical challenges and limitations inherent to dental FEA. A narrative literature search was conducted across PubMed/MEDLINE, Scopus, EMBASE, the Cochrane Library, and Google Scholar using the terms “finite element analysis,” “FEA,” “dental biomechanics,” and “stress distribution,” combined with specialty-specific MeSH headings. Articles published in English from 1969 to June 2026 were considered. Inclusion criteria were primary FEA research articles, systematic reviews, and authoritative biomechanics textbooks. Exclusion criteria were non-English language publications, conference abstracts without full-text availability, and studies involving non-dental anatomical sites exclusively. No formal PRISMA-based systematic process was applied, consistent with the narrative review format; this review was not prospectively registered. FEA has demonstrated substantial methodological utility as a hypothesis-generating and design-optimisation tool across implantology, orthodontics, endodontics, prosthodontics, periodontics, oral and maxillofacial surgery, restorative dentistry, paediatric dentistry, and temporomandibular joint disorders; however, much of the available literature remains computational and hypothesis-generating rather than clinically validated. Key applications include peri-implant stress distribution modelling, orthodontic force optimisation, endodontic instrument fatigue prediction, and prosthetic material selection. Persistent limitations include oversimplification of biological tissue properties, absence of validated dynamic loading models, and lack of inter-study standardisation. The quality of FEA outputs is critically dependent on the accuracy of geometry reconstruction, the completeness of material property assignment, the realism of loading conditions, and the presence of experimental model validation. FEA is a valuable tool in evidence-based dental research, enabling non-invasive, patient-specific biomechanical simulation. Advances in CBCT imaging, AI-assisted meshing, and high-performance computing are progressively addressing existing limitations and expanding clinical translatability.

## 1 Introduction

Dentistry, once rooted primarily in craftsmanship and clinical artistry, is undergoing a technological renaissance fuelled by biomechanics and computational simulation. Among the most transformative tools in this new era is Finite Element Analysis (FEA), a numerical technique originally pioneered in aerospace and civil engineering in the mid-twentieth century and subsequently adapted to biomedical sciences for analysing complex structures including bones, teeth, and prosthetic systems (1, 2).

The oral cavity presents a uniquely challenging biomechanical environment, characterised by irregular three-dimensional geometries, heterogeneous and anisotropic materials, and complex multiaxial load dynamics arising from mastication, parafunction, and orthodontic appliances. Traditional experimental methods such as photoelasticity and strain-gauge measurements, though informative, carry notable limitations as they provide either qualitative insights or surface-localised data that cannot capture internal stress gradients (3, 4). FEA, by contrast, offers quantitative, three-dimensional simulation of the entire structural domain which includes subsurface regions inaccessible to conventional measurement without requiring physical specimens or invasive procedures (5–7).

Since Branemark's seminal 1969 description of intra-osseous anchorage and the subsequent development of the osseointegration concept (8), the long-term success of dental implants has been understood to depend critically on biomechanical harmony between the prosthesis, bone, and applied occlusal forces. FEA emerged as a key bridge in this context, enabling researchers to evaluate prosthetic designs, optimise implant placement geometry, model bone remodelling under cyclic loading, and anticipate long-term treatment outcomes (6, 9). With the advent of cone-beam computed tomography (CBCT), it is now feasible to generate patient-specific FEA models that approximate patient-specific geometry and, where validated against calibration data, regional bone density distributions (10, 11).

Today, FEA applications in dentistry extend far beyond implantology, encompassing orthodontics, prosthodontics, endodontics, periodontics, restorative dentistry, paediatric dentistry, and oral and maxillofacial surgery. It must be noted, however, that despite the exponential growth of FEA publications over the past two decades, a substantial proportion of computational findings have not undergone prospective clinical validation, and predictive reliability varies markedly with the modelling assumptions applied. Furthermore, while CBCT-derived patient-specific models represent a significant advance in anatomical fidelity, they still require substantial simplifications in material property assignment, interface behaviour definition, and loading representation; anatomical accuracy should not be conflated with full biological representativeness (10, 11). This narrative review addresses a gap in the existing literature by integrating historical, methodological, multi-specialty, and limitation-focused content within a single unified framework accessible to both dental clinicians and computational researchers, providing a structured critical appraisal from historical foundations and methodological framework through specialty-specific applications to persistent technical challenges and future directions. Existing reviews of FEA in dentistry remain narrower in scope or evidentiary depth. Falcinelli et al. (2023) confine their critical appraisal to implant dentistry alone (12). Shivakumar et al. (2021) (13) survey FEA across several specialties but draw on only 44 included studies weighted predominantly toward implant applications, without a dedicated analysis of inter-study methodological heterogeneity, a quantitative evidence map of validation status by specialty, or a structured taxonomy of FEA's biology-inherent limitations (13). The present review combines all-specialty coverage with an explicit methodological-heterogeneity analysis (Section 5.5.1), a cross-specialty evidence map (Table 1), and a seven-point taxonomy of fundamental limitations (Section 6.1).

**Table 1:** Evidence map of methodological quality and validation status of dental FEA literature, by specialty.

Specialty	Representative systematic/scoping review	Studies included	Validation/methodological quality finding
Implantology and Prosthodontics	Qiu et al. (2024) (47), implant length/diameter; historical benchmark Chang et al. (2018) (42)	40 (of 852 screened); 601 (1997–2016) for the historical benchmark	Only 1/40 (2.5%) studies experimentally validated the FE model; 11/40 reported convergence testing. Chang et al. found 48/601 (8%) incorporated any validation, with high-level *in vivo* human validation in a single study.
Endodontics	Chien et al. (2024) (107), extended finite element method (XFEM) in endodontics	4 (of 13 screened)	No study to date has applied XFEM to cyclic fatigue or crack propagation testing of NiTi rotary instruments; the authors attribute this to challenges in modelling material inputs and fatigue criteria.
Orthodontics	Cao et al. (2025) (60), clear aligner biomechanics	29	All 29 included studies graded only moderate (“B”) referential reliability; no unified protocol exists for FEA model setup or data presentation in clear aligner research.
Periodontics	No dedicated FEA methodological-quality review identified; closest evidence: Ruse (2008) (109) and Dhammayannarangsi et al. (2025) (93)	Not applicable (narrative/sensitivity analyses, not systematic reviews)	Erroneous periodontal ligament elastic-modulus values introduced into the literature in 1980 have propagated through hundreds of FEM/FEA papers, with errors of two to three orders of magnitude (109). A controlled sensitivity analysis found that an inappropriately low value overestimates stress by up to 1195% (93).
Restorative Dentistry	Richert et al. (2020) (103), validated FE models of premolars	19 (of 214 full-text-screened, from 1306 records)	Only 25/214 (11.6%) full-text articles presented a validated FE model; 136/214 described no validation process at all, and a further 53 used convergence testing or unquantified mechanical comparison only.
Oral and Maxillofacial Surgery	Daqiq et al. (2025) (105), mandible input parameters; Soodmand et al. (2024) (106), mandible material models	66 (of 13,023 screened); 77 (material-model review)	Daqiq et al. admitted only *in vivo* bite-force data and assessed risk of bias with validated NIH checklists. Soodmand et al. identified 12 distinct heterogeneous bone material models via the dentistry-specific ROBFEAD tool, rating original studies low-to-medium risk of bias.
Temporomandibular Joint Disorders and Bruxism	No dedicated FEA methodological-quality review identified; closest evidence: Rodrigues et al. (2018) (108), confined to TMJ replacement devices	19 (of 307 screened)	Scope limited to TMJ replacement (TMJR) device biomechanics; does not address native joint, disc, or bruxism-related loading, and reports no validation-rate or risk-of-bias statistic.
Paediatric Dentistry	Atif et al. (2024) (104), dental trauma FEA	46 (2001–2023)	All 46 included studies (100%) rated high risk of bias via the ROBFEAD tool (inter-rater Cohen's kappa 0.962); maxillary central incisors were the most modelled structure (27/46).

A fundamental caveat applies across all specialty applications surveyed in this review: FEA functions as a hypothesis-generating and design-optimisation tool, not as a direct source of clinical evidence. Its outputs quantify mechanical stress and strain under explicitly defined and often simplified modelling assumptions; they do not constitute clinical proof of treatment efficacy or device safety. Translation from computational findings to clinical recommendations requires prospective experimental or clinical validation under conditions that correspond to those of the model. This distinction is maintained throughout the review and is addressed in detail in Sections 5 and 6.

## 2 Methods: literature search strategy

This narrative review was conducted through electronic searches of PubMed/MEDLINE, Scopus, EMBASE, the Cochrane Library, and Google Scholar. The following Medical Subject Headings (MeSH) and free-text terms were used in combination: “finite element analysis,” “FEA,” “finite element method,” “dental biomechanics,” “stress distribution,” “bone remodelling,” and specialty-specific terms including “implantology,” “orthodontics,” “endodontics,” “prosthodontics,” “periodontics,” “oral surgery,” “restorative dentistry,” “temporomandibular joint,” and “paediatric dentistry.” Boolean operators (AND, OR) were applied to combine concepts; no date filter was applied during initial searching.

### Articles published in English from 1969 to June 2026 were eligible

The primary Boolean search string applied in PubMed/MEDLINE was: ([“finite element analysis"[MeSH Terms] OR “finite element method"(All Fields) OR “FEA"(All Fields)] AND (“dental biomechanics"(All Fields) OR “stress distribution"(All Fields) OR “implantology"(All Fields) OR “orthodontics"[MeSH Terms] OR “endodontics"[MeSH Terms]OR “prosthodontics"[MeSH Terms]OR “periodontics"[MeSH Terms]OR “temporomandibular joint"[MeSH Terms]OR “paediatric dentistry"(All Fields))). Analogous field-adapted syntax was applied in Scopus (TITLE-ABS-KEY fields), EMBASE (Emtree terms and free-text), the Cochrane Library (title, abstract, and keyword fields), and Google Scholar. Searches were last updated in June 2026.

Inclusion criteria were: (1) primary FEA research articles reporting dental or craniofacial biomechanical outcomes; (2) systematic reviews and meta-analyses of dental FEA literature; and (3) authoritative engineering and biomechanics textbooks providing foundational methodological context.

Exclusion criteria were non-English language publications; conference abstracts without full-text availability; and studies exclusively investigating non-dental anatomical sites. Reference lists of included articles were manually screened for additional relevant sources. As this is a narrative and not a systematic review, no formal PRISMA flow diagram is presented.

Within each thematic section, study selection prioritised landmark publications with high citation impact, investigations incorporating experimental validation, patient-specific geometric reconstruction, or nonlinear material modelling, and methodologically robust representative studies across each specialty domain. Where multiple studies of comparable methodological quality addressed similar research questions, more recent publications were preferentially included.

Seminal studies were retained to provide historical and conceptual context, alongside contemporary investigations employing CBCT-based modelling approaches. Accordingly, the cited literature should be interpreted as a curated, representative synthesis rather than an exhaustive compilation of all available studies.

Additionally, two relevant publications identified during the peer-review revision process [Ausiello et al., 2026 (14) and Borges et al., 2025 (15)] were incorporated as post-search inclusions. Although not retrieved in the initial database search, these studies were considered directly pertinent to the restorative dentistry domain and were included to strengthen the methodological discussion presented in Section 5.5.1.

## 3 Historical development of FEA: A timeline

### 3.1 Early theoretical foundations (Pre-1940s)

The mathematical basis for FEA emerged from early numerical approximation studies in structural mechanics. In the eighteenth century, Euler and Bernoulli formulated beam theory that would later become central to structural analysis (16). In 1943, Courant introduced the use of piecewise polynomial interpolation for solving variational problems, now recognised as an early conceptual precursor to the finite element method (17).

### 3.2 Initial development (1940s to 1950s)

Hrennikoff (1941) developed the framework method for solving elasticity problems by discretising continuous domains (18). Turner, Clough, Martin, and Topp (1956) published a landmark paper in the Journal of the Aeronautical Sciences formally introducing stiffness matrix methods for structural analysis, which is widely credited as the earliest formal use of what would become the finite element method (19). Clough subsequently coined the specific term “finite element” in a paper presented at the 2nd ASCE Conference on Electronic Computation in Pittsburgh in 1960 (20).

### 3.3 Expansion and computerisation (1960s to 1970s)

Computational advances drove rapid growth of FEA in the 1960s. Argyris and Zienkiewicz integrated matrix methods with finite element formulations, enabling fully computerised solutions (21). NASA's development of the NASTRAN finite element solver under contract in the late 1960s, documented by MacNeal (1989) and Schaeffer (2001), significantly boosted FEA's scientific credibility and spurred the release of dedicated commercial packages (22, 23). By the 1970s, NASTRAN, ANSYS, and ABAQUS had been released, establishing FEA as an industry standard in engineering (24).

### 3.4 Biomedical and dental adaptation (1970s to 2000s)

FEA was first applied to dental structures in the early 1970s. Farah, Craig, and Sikarskie (1973) were among the earliest investigators to apply FEA to dental restorations, examining stress distribution in a restored axisymmetric first molar (1). Three-dimensional dental FEA models became feasible through the 1980s and 1990s as computational power grew. Patient-specific modelling using CT data emerged in the 2000s, dramatically improving model anatomical fidelity (10, 11).

### 3.5 Modern computational Era (2000s to present)

Contemporary FEA in dentistry benefits from integration with artificial intelligence for automated meshing, cloud-based high-performance computing (HPC), and direct import of patient CBCT datasets into modelling environments. Advanced computational techniques now allow the use of nonlinear material models, simulations involving dynamic loading, and multiphysics analyses that integrate mechanical, thermal, and biological responses within a single model (25).

## 4 Finite element analysis: methodological framework

**Figure 1:**
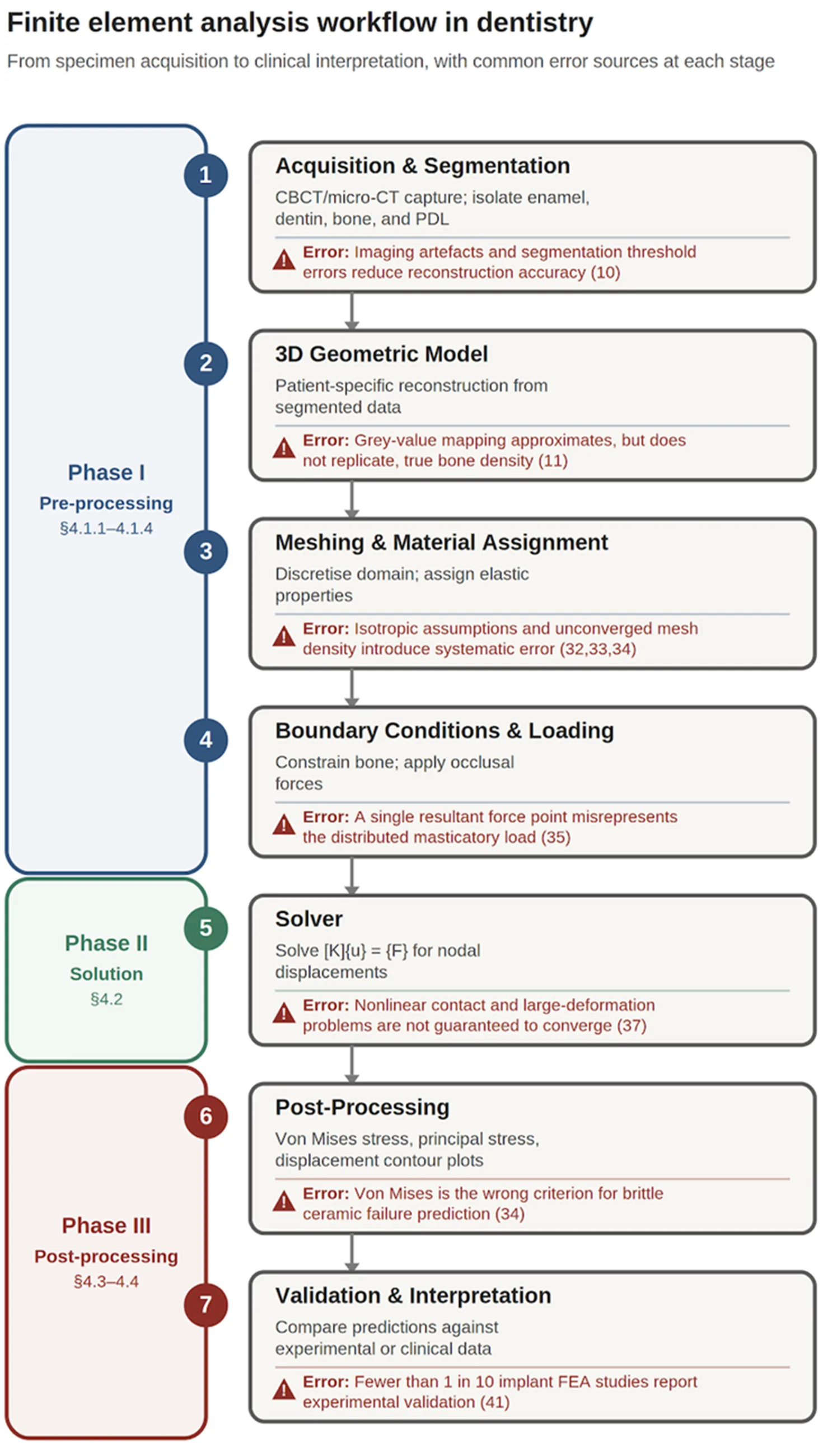
Workflow of finite element analysis in dentistry, from specimen acquisition to clinical interpretation, organised across the pre-processing, solution, and post-processing phases (section 4.1–4.4).

FEA involves the discretisation of a complex geometric domain into a finite number of simpler sub-domains (elements), within which the governing physical equations are solved numerically. In dentistry, this process is systematically divided into three phases: pre-processing, solution, and post-processing (Figure 1) (26).

### 4.1 Pre-Processing phase

#### 4.1.1 Geometric model generation

The geometric model of the dental structure of interest is either created using CAD software or, more commonly in dental research, reconstructed from CBCT or micro-CT imaging data using segmentation software (e.g., Mimics, 3-matic, ITK-SNAP). The accuracy of this reconstruction directly determines the anatomical fidelity of the simulation (10). Patient-specific models derived from CBCT data allow individualised bone density distributions to be incorporated via grey-value mapping (11).

#### 4.1.2 Material property assignment

Each component of the model such as enamel, dentin, periodontal ligament (PDL), cortical bone, cancellous bone, implant and restoration is assigned appropriate material properties. The most fundamental parameters are Young's modulus (elastic stiffness) and Poisson's ratio (lateral strain response).

 Table 2 summarises validated material properties for key dental tissues and materials, drawn from primary mechanical characterisation studies (27–33, 110).

**Table 2:** Representative material properties of dental tissues and materials used in dental FEA modelling.

Material/Tissue	Young's Modulus (GPa)	Poisson's Ratio	References
Enamel	80–84	0.30	(27)
Dentin	18–20	0.31	(28)
Cortical Bone	13–17	0.30	(11)
Cancellous Bone	0.5–1.5	0.30	(11)
Periodontal Ligament	0.05–0.07	0.45	(5)
Titanium (implant)	110–120	0.35	(110)
Zirconia (crown)	200–210	0.31	(30, 31)
Composite Resin	10–20	0.24	(32)
PMMA (denture base)	2.5–3.5	0.35	(33)

A critical methodological consideration is whether tissues are modelled as isotropic (same properties in all directions), orthotropic, or anisotropic. Biological tissues such as bone and enamel are inherently anisotropic; however, isotropic assumptions are commonly made for computational simplicity. Such simplifications must be explicitly acknowledged as potential sources of systematic error and should ideally be addressed through sensitivity analyses (13, 34).

#### 4.1.3 Meshing

The continuous geometric structure is divided into a mesh composed of finite elements, typically hexahedral (brick) or tetrahedral elements in three-dimensional dental models. The quality of the mesh is crucial, as meshes that are too coarse can produce numerical inaccuracies, whereas extremely fine meshes increase computational demands without providing proportional improvements in accuracy. Mesh convergence studies, where the mesh density is gradually refined until the results stabilise within a predetermined tolerance, are regarded as an essential step for validating the model and should be reported in published research (35).

#### 4.1.4 Boundary conditions and loading

Boundary conditions define how the structure is supported (constraints) and loaded (applied forces). In dental FEA, the bone is typically constrained at its periphery, and occlusal forces are applied at defined locations on the crown or implant. The direction, magnitude, and point of application of forces critically influence stress distributions. It is important to distinguish between habitual masticatory forces which range from approximately 50 N (incisors) to 200 N (molars) during normal function and maximum voluntary bite forces, which may reach 400–800 N in adult subjects with high masticatory muscle mass (36). Selection of appropriate force values must be justified by reference to *in vivo* bite-force studies and clearly defined in the methods of any publication.

### 4.2 Solution phase

The assembled system of equations expressed in matrix form as (K){u} = {F}, where (K) is the global stiffness matrix, {u} is the nodal displacement vector, and {F} is the applied force vector, is solved using either direct solvers (e.g., Gaussian elimination) or iterative solvers (e.g., Conjugate Gradient method) (37). For nonlinear analyses (e.g., large deformations, contact problems, viscoelastic PDL), iterative Newton-Raphson methods are employed (38). Convergence criteria and solver tolerances must be stated in any published study.

### 4.3 Post-Processing phase

Results are visualised as contour plots of von Mises stress, principal stresses, shear stress, or nodal displacement. Von Mises stress is the most widely reported metric in dental FEA, providing a scalar measure suitable for failure prediction in ductile materials; for brittle materials such as ceramics, maximum principal (tensile) stress is the more appropriate criterion (35). Results must be interpreted in the context of each material's ultimate tensile or compressive strength, and comparisons between models are only valid when boundary conditions and material properties are matched (39).

### 4.4 Validation

Model validation is a mandatory step that is frequently underreported in dental FEA literature. Validation involves comparing FEA-predicted strains or displacements against experimentally measured values from strain gauges, digital image correlation, or photoelastic studies on physical replicas (40). Authors are strongly encouraged to report validation data or, where *in vitro* validation is not feasible, to perform sensitivity analyses examining how results change with variations in key input parameters. Two foundational frameworks should be consulted: ASME V&V 40-2018, which defines minimum credibility standards for computational modelling in medical device development; and Erdemir et al. (2012), which provides discipline-specific minimum reporting requirements for FEA studies in biomechanics (40, 41). Adherence to these standards is increasingly expected by high-impact journals. The extent of the validation deficit in the existing dental FEA literature has been directly quantified: Chang et al. (2018), in a systematic review of 601 dental implant FEA articles published between 1997 and 2016, found that only 48 (8%) incorporated any form of model validation, with high-level *in vivo* validation using human subjects present in a single study (42). This figure is representative of the broader pattern across dental FEA specialties and underscores that most published computational findings in dentistry rest on unvalidated models, a limitation that substantially constrains their clinical interpretability.

## 5 Applications of FEA in dentistry

### 5.1 FEA in implantology and prosthodontics

Implantology represents the most extensively studied domain of dental FEA, with a literature dating to the early 1970s (1). FEA is used to evaluate how occlusal forces are transmitted through the implant-abutment-restoration complex to the surrounding bone, with the goal of identifying stress concentrations that may predispose to bone resorption or implant failure (43).

Several biomechanical principles have been consistently supported across multiple independent FEA studies: cortical bone at the implant neck is the site of maximum stress concentration; and off-axis (non-axial) loading significantly increases peri-implant bone stress compared to axial loading (43, 44). However, it is important to note that some findings remain contested. The assertion that the implant diameter has greater influence on crestal bone stress than implant length is model-dependent. Petrie and Williams (2005) demonstrated that the relative influence of diameter and length varies significantly with bone density and loading angle, and results should not be generalised across all clinical scenarios (45). Similarly, platform switching has been modelled as reducing peri-implant bone stress concentrations (44), but subsequent FEA studies using different implant geometries have produced conflicting results, underscoring the need for study-specific interpretation rather than universal conclusions (43). FEA has also been used to compare one-piece vs. two-piece implant systems, evaluate tilted implant configurations (e.g., All-on-4), and optimise abutment designs (46). Recent systematic reviews have further characterised the differential effects of implant length and diameter on crestal bone stress across bone quality categories (47).

The apparent contradictions among implantology FEA studies described above are not random inconsistencies but predictable consequences of systematic modelling differences. Cortical bone thickness assumptions, the degree and spatial distribution of modelled osseointegration (fully bonded vs. frictional contact), loading direction relative to the implant axis, bone quality category (Type I through Type IV), and crown-to-implant ratio all independently modify peri-implant stress outputs (43, 45). A study reporting that wider implants reduce crestal bone stress may have modelled only axial loading in Type II bone with full osseointegration; the same geometry under off-axis loading in Type IV bone with partial osseointegration may yield the opposite conclusion. This dependency on input assumptions means that implantology FEA findings are model-specific rather than universally generalisable, and clinical extrapolation requires careful consideration of whether a study's modelling conditions match the clinical scenario in question.

In prosthodontics, finite element analysis has been used to evaluate stress distribution and fracture risk in prostheses made from metal, ceramic materials such as zirconia and lithium disilicate, and polymer-based materials. Studies using FEA have shown that all-ceramic crowns tend to develop tensile stresses at the cementation interface and within the ceramic core, observations that have contributed to recommendations regarding optimal layer thickness (48). Removable prosthesis design, including implant-supported overdentures, has also been optimised using FEA by examining load distribution across abutments and mucosal support (49).

### 5.2 FEA in endodontics

In endodontics, FEA has been applied to analyse stress distribution in root canal-treated teeth, evaluate the mechanical behaviour of nickel-titanium (NiTi) rotary instruments, assess fracture resistance of post-and-core systems, and simulate obturation and irrigation forces (50).

Stress analysis has demonstrated that endodontically treated teeth are particularly susceptible to vertical root fracture at the mid-root level and at thin dentin walls following canal preparation, with stress concentrations amplified by tapered preparations and excessive irrigation pressures (50, 51). FEA studies of NiTi rotary files have characterised the relationship between file taper, cross-sectional geometry, and cyclic fatigue, findings that have contributed to design improvements in file systems (52). The comparative behaviour of fibre posts and cast metal posts in endodontically treated teeth has been widely investigated using finite element models. These studies indicate that fibre posts tend to produce a more uniform stress distribution that closely matches the elastic modulus of dentin, thereby lowering the likelihood of catastrophic root fracture (53).

Thermal analysis during endodontic procedures has also been modelled using FEA. The critical threshold for heat-induced bone injury is an increase of 10 degrees C above physiological temperature, as established by Eriksson and Albrektsson (1983) in a vital-microscopic study (54). Studies examining heat generation during warm vertical compaction obturation support the use of intermittent application protocols to remain below this threshold. The effects of irrigant solutions, including sodium hypochlorite, on NiTi file mechanical properties and fracture susceptibility have also been characterised experimentally, with findings that directly inform file design guidelines (55).

It should be emphasised that the endodontic FEA findings described above are model-dependent. Predictions of root fracture susceptibility and stress concentration patterns are sensitive to root morphology, remaining dentin wall thickness at the critical cross-section, canal preparation taper, and the specific loading magnitude and direction applied. Studies modelling the same clinical scenario but varying these parameters can produce substantially different fracture risk estimates. Endodontic FEA findings should therefore be interpreted as directional indicators of relative risk rather than absolute mechanical thresholds, and clinical application requires verification against experimental fracture studies conducted under matched conditions.

### 5.3 FEA in orthodontics

Orthodontic FEA focuses on tooth movement mechanics, force distribution through the periodontal ligament (PDL), alveolar bone stress, and appliance design optimisation (56).

Key applications include prediction of tooth displacement trajectories under orthodontic force systems, which has informed the design of bracket systems and clear aligner attachment geometries (57). However, a critical limitation of all current clear aligner FEA models deserves explicit statement: these simulations model only the initial mechanical response of the periodontal ligament and surrounding alveolar bone at the instant of force application (57). They do not represent the biological remodelling processes, including osteoclastic resorption at the pressure side and osteoblastic apposition at the tension side, governed by the strain-regulated mechanostat described by Frost (58), that are the actual cellular mechanism of tooth movement in clinical practice. More recent analyses have extended this work to extraction space closure under different anchorage controls, providing more clinically representative modelling of staged aligner biomechanics (59). A systematic review of 29 FEA studies on clear aligner biomechanics confirmed that no unified protocol currently exists for model setup or data presentation in aligner research, and that published studies consistently address only the initial instantaneous mechanical response rather than the long-term biomechanical effects of staged aligner treatment (60). Nonetheless, FEA-derived predictions regarding aligner force systems should not be interpreted as predictive of clinical tooth movement trajectories or treatment timelines, as these are determined by biological adaptation responses that no current structural FEA framework can simulate. FEA studies of PDL stress distribution have elucidated the biomechanical basis of root resorption: excessive compressive stress in the PDL at the root apex under sustained tipping forces is associated with increased resorption risk (61). Miniscrew anchorage (TAD) placement optimisation has been investigated using three-dimensional FEA to quantify bone stress generated at the insertion site relative to adjacent root proximity (62). Rapid maxillary expansion (RME) has been modelled extensively, revealing suture opening patterns and stress distributions in the mid-palatal suture that depend on patient age and skeletal maturity (63).

### 5.4 FEA in periodontics

Periodontal FEA models the biomechanical response of the supporting apparatus such as alveolar bone, PDL, and cementum under masticatory, orthodontic, and traumatic loading (64).

FEA has identified that alveolar bone stress concentrations at the crest are amplified in the presence of angular bone defects, providing a biomechanical rationale for the progressive nature of bone loss in periodontitis (64). Geramy (2002) demonstrated that increasing levels of alveolar bone loss progressively alter PDL stress distribution under orthodontic loading, with clinical implications for treatment planning in periodontally compromised patients (64). The influence of occlusal trauma on periodontal tissues has been modelled, demonstrating that excessive parafunctional forces significantly increase PDL compressive stress, particularly in teeth with reduced periodontal support, and may accelerate alveolar bone resorption (65). FEA has also been applied to evaluate regenerative interventions, modelling how bone grafts and resorbable membrane placements alter the biomechanical environment of treated defects, with implications for membrane design and graft stability (66).

### 5.5 FEA in restorative dentistry

Restorative FEA evaluates the mechanical performance of cavity preparations, direct and indirect restorative materials, and adhesive interfaces under occlusal loading (67).

Composite resin FEA studies have characterised the influence of cavity design on polymerisation shrinkage stress and subsequent interfacial debonding. The C-factor (ratio of bonded to unbonded surfaces) has been validated as a critical predictor of shrinkage stress in Class I vs. Class II preparations (68). Variations in curing technique (bulk-fill vs. incremental layering), filler particle size, and elastic modulus of composites have been modelled to assess their effect on internal stress gradients and gap formation. El-Damanhoury and Platt (2014) demonstrated that bulk-fill composites exhibit reduced polymerisation shrinkage stress kinetics compared to conventional incrementally placed composites, with implications for cavity design (69).

Ceramic crown FEA has delineated the relationship between ceramic layer thickness, core design, and fracture probability, informing minimum thickness guidelines for posterior zirconia restorations (48). Magne and Belser (2003) demonstrated using FEA that porcelain inlays and onlays generate measurably different stress distributions and crown flexure profiles compared to composite restorations under equivalent loading, findings that inform adhesive restoration selection for posterior teeth (67). The comparative performance of cast metal post-and-core systems vs. fibre post systems has also been extensively studied. FEA studies indicate that cast metal posts generate higher root stress concentrations than fibre post systems, findings that have contributed to the clinical preference for adhesive fibre post systems in structurally compromised teeth (53). Marghalani et al. (2012) extended this analysis to custom-made CAD/CAM zirconia dowels, demonstrating through three-dimensional FEA that zirconia post-and-core restorations produce stress distributions comparable to cast gold and represent a viable aesthetic alternative in endodontically treated teeth (70). Awad (2003) investigated the stress distribution generated by resin luting cements of varying thickness under ceramic restorations, findings that contribute to evidence-based luting cement selection for all-ceramic indirect restorations (71).

#### 5.5.1 Methodological heterogeneity in restorative FEA

A dedicated examination of methodological heterogeneity is warranted in restorative FEA because diversity in modelling assumptions represents a dominant source of inter-study variability that can exceed the actual biomechanical differences under investigation. Ausiello et al. (2026), in a parallel narrative review specifically addressing FEA methodological validity in polymer-based adhesive restorations, identified simplified material models, idealised bonding conditions, and static loading protocols as the principal constraints limiting the clinical interpretability of published restorative FEA findings (14).

Six sources of heterogeneity merit specific discussion. First, bonded vs. non-bonded interface assumptions: most restorative models apply either a perfectly bonded adhesive interface or zero bonding (frictionless contact); neither represents the clinical hybrid layer, where incomplete resin tag penetration, interfacial porosity, and progressive water sorption introduce local mechanical discontinuities that alter marginal stress in ways no idealised interface model can reproduce.

Second, linear vs. nonlinear material modelling: composite resins exhibit viscoelastic creep and stress relaxation under sustained loading, behaviours absent from linear-elastic frameworks and representing a systematic source of long-term prediction error.

Third, polymerisation shrinkage simulation method: some studies apply shrinkage as a thermal analogy, others as a pre-strain, and others omit it entirely, generating fundamentally incomparable stress magnitudes across studies that nominally address the same restorative question. Borges et al. (2025) demonstrated in a 3D FEA study of Class III restorations that the choice of restorative composite combination significantly alters internal shrinkage stress distribution and that the bevel area at the cervical margin consistently concentrates the highest shrinkage stress regardless of composite system (15). This finding has direct implications for marginal integrity and illustrates how shrinkage simulation methodology influences clinically relevant outcomes.

Fourth, thermal cycling: intraoral temperature fluctuations between 5 and 60 degrees C generate cyclic interfacial stresses comparable in magnitude to occlusal loading stresses (72), yet most restorative FEA studies model the system at a single temperature.

Fifth, fatigue framework absence: reporting peak stress under a single load application without applying S-N curves or fracture mechanics frameworks makes it impossible to assess whether margins or ceramic layers will remain intact across millions of masticatory loading cycles.

Sixth, operator-dependent meshing choices in regions with high stress gradients, such as adhesive interfaces and cavity margin angles, independently alter reported stress peak magnitudes. Without formal sensitivity analyses, differences between published restorative FEA studies cannot be reliably attributed to material or cavity design effects rather than modelling decisions.

### 5.6 FEA in oral and maxillofacial surgery

In oral and maxillofacial surgery, FEA is used to optimise fixation hardware design, plan reconstructive procedures, simulate craniofacial trauma, and evaluate distraction osteogenesis outcomes (73).

Mandibular fracture fixation has been extensively modelled, comparing single-plate vs. double-plate osteosynthesis at the mandibular angle. FEA results indicate that two-plate fixation provides superior stress distribution and reduced risk of hardware failure, consistent with clinical evidence (74). Experimental and finite element studies of the human mandible under blunt impact loading have characterised fracture susceptibility patterns and the relationship between impact direction and fracture site (75). Distraction osteogenesis protocols have been analysed using FEA by Carter et al. (1998), who characterised the mechanobiological environment at the regenerate site and identified optimal mechanical conditions to promote intramembranous ossification (76). Orthognathic surgical outcomes, including bilateral sagittal split osteotomies, have been modelled using FEA to predict post-surgical changes in occlusal force distribution, condylar loading, and skeletal displacement, providing pre-operative planning support (77).

### 5.7 FEA in temporomandibular joint disorders and bruxism

The temporomandibular joint (TMJ) presents one of the most biomechanically complex modelling challenges in dentistry due to the disc, condyle, fossa, and associated musculature acting as an integrated system (78). The articular disc is particularly challenging to model because it exhibits biphasic, viscoelastic, and nearly incompressible behaviour under load, which standard isotropic FEA material models fail to capture accurately. Most TMJ finite element analyses simplify the disc as either linearly elastic or hyperelastic, leading to systematic errors in estimating condylar contact stresses. In addition, synovial fluid dynamics and joint lubrication cannot be represented by structural FEA alone, requiring fluid-structure interaction approaches that remain computationally demanding (78, 79).

Three-dimensional FEA models of the TMJ have characterised condylar stress distribution during mandibular opening, lateral excursion, and clenching, demonstrating that disc displacement significantly alters condylar loading patterns (79). Sagl et al. (2022) used an in-silico musculoskeletal model incorporating dynamic TMJ loading to demonstrate that bruxism-related facet inclination and contact location generate von Mises stress in the condylar cortex that may exceed the fatigue threshold of cortical bone under sustained parafunctional activity (80). Occlusal splint therapy has been modelled, demonstrating that stabilisation splints effectively redistribute and reduce condylar compressive stress, providing biomechanical evidence for their clinical application (78). Mandibular advancement device (MAD) design for obstructive sleep apnea management has been evaluated using FEA, examining how advancement magnitude and device stiffness affect TMJ loading (81). Total joint prosthesis design has been evaluated using FEA to predict condylar and fossa stress distribution under simulated functional loads, informing prosthetic geometry (82).

### 5.8 FEA in paediatric dentistry

FEA applications in paediatric dentistry have focused on traumatic dental injuries, cleft lip and palate management, and craniofacial growth modification. The paediatric craniofacial skeleton with its developing bone, primary dentition, and mixed dentition presents unique biomechanical properties that differ from adult models, necessitating age-specific material property assignment (13).

Mouthguard effectiveness in protecting maxillary incisors from traumatic impact has been modelled using FEA, demonstrating that custom-fabricated, dual-laminated mouthguards significantly reduce transmitted peak force compared to stock variants (83). Stress distribution during facemask protraction therapy in cleft lip and palate patients has been modelled using patient-specific CBCT data, revealing asymmetric displacement patterns at the mid-palatal suture that are clinically relevant for treatment planning (84). Pre-surgical nasoalveolar moulding (NAM) appliance design has been evaluated using FEA to optimise pre-surgical soft tissue shaping forces in infants with complete clefts, with three-dimensional analysis confirming differential tissue displacement between conventional and modified appliance configurations (85).

## 6 Technical challenges in dental FEA

Despite its considerable utility, finite element analysis (FEA) in dentistry is associated with several methodological challenges that warrant explicit recognition and transparent reporting. These limitations are not independent of the clinical applications discussed in Sections 5.1 through 5.8; rather, they directly influence the interpretation and generalisability of findings across different domains.

In implantology (Section 5.1), the common assumptions of isotropic bone properties and perfectly bonded implant–bone interfaces may lead to inaccurate representation of peri-implant stress distributions in heterogeneous biological environments. In endodontics (Section 5.2), the absence of fatigue modelling restricts interpretation, as stress values derived under single static loading conditions do not account for cumulative damage under cyclic functional loading. In orthodontics (Section 5.3), models typically simulate only the initial periodontal ligament (PDL) response, thereby limiting their ability to predict long-term tooth movement. Similarly, in restorative dentistry (Section 5.5), reliance on static loading conditions, idealised bonding assumptions, and single-temperature environments constrains clinical extrapolation, as elaborated in Section 5.5.1.

Accordingly, interpretation of FEA outcomes across dental specialties should be undertaken with consideration of the relevant methodological constraints outlined in Table 3 and Sections 6.1.1 through 6.1.7. Table 3 summarises the principal limitation domains, their methodological basis, and their associated clinical or scientific implications. These limitations are broadly categorised into two groups: (i) addressable limitations (Rows 1–9), which may be mitigated through advances in experimental validation, computational modelling, and standardisation; and (ii) inherent methodological limitations (Rows 10–16), which arise from the fundamental constraints of applying structural FEA to complex, living biological systems and cannot be fully resolved through *in vitro* or computational approaches alone.

**Table 3:** Technical challenges in dental FEA.

No.	Domain	Issue	Challenge/Impact
1	Complex Geometry	Teeth, PDL, bone, and restorations have highly irregular 3D shapes	Accurate meshing of anatomically complex structures is time-consuming and technically demanding (36)
2	Material Properties	Biological tissues are anisotropic, viscoelastic, and heterogeneous	Isotropic assumptions reduce model reliability and predictive accuracy (65)
3	Boundary Conditions	Masticatory forces vary in direction, magnitude, and duration	Realistic constraint simulation is difficult to replicate precisely; habitual and maximum forces must be distinguished (37)
4	PDL Modelling	PDL exhibits non-linear, time-dependent behaviour under load	Most models oversimplify the PDL, affecting tooth-mobility and stress-transmission predictions (38)
5	Dynamic vs. Static	Most FEA models use static loading; mastication involves cyclic dynamic loads	Dynamic simulation requires more complex models and greater computational resources (40)
6	Computational Cost	High-fidelity FEA demands large processing power and time	Long runtimes hinder routine clinical adoption (41)
7	Validation	*In vivo* validation data are scarce	Without experimental validation against ASME V&V 40 standards and Erdemir et al. (2012) reporting guidelines, predictive reliability remains unconfirmed (35, 39)
8	Mesh Quality	Poor meshing leads to numerical inaccuracies and non-convergence	High-quality meshes are essential but labour-intensive to generate (43)
9	Standardisation	No universal guidelines exist for dental FEA modelling protocols	Inconsistencies in model setup, material properties, and validation methods limit inter-study comparability (44)
10	Biological Irreducibility	FEA models tissues as inert solids; bone remodeling, mechanotransduction, PDL fluid dynamics, and immune responses are entirely absent	Long-term biological predictions (peri-implant remodeling, orthodontic bone changes) carry inherent uncertainty that no *in vitro* study can resolve (86, 95)
11	Implant-Bone Interface Assumption	Interface modelled as fully bonded or frictionless; neither reflects the spatially heterogeneous osseointegration continuum	Critically affects peri-implant stress in immediate loading and poor bone quality scenarios; micromotion-driven osseointegration failure cannot be predicted (87)
12	Muscle Force Oversimplification	Masticatory loading reduced to a single resultant force; the coordinated, time-varying, 3D muscle force system cannot be uniquely determined from anatomy alone	Systematic underestimation or mislocalisation of stress peaks, particularly in TMJ, bruxism, and All-on-4 models (88)
13	Residual Stress, Thermal Cycling and Creep	Fabrication residuals, intraoral thermal fluctuations (5–60 degrees C), and time-dependent creep in PMMA and PDL are routinely excluded from models	Underestimates fracture risk in ceramic restorations and removable prostheses; thermal stresses are comparable in magnitude to occlusal stresses (72)
14	Fatigue Life Prediction	Standard FEA outputs single-load peak stress; S-N curves, Paris law crack propagation, and Weibull fracture frameworks needed to predict cyclic fatigue life are absent in most dental studies	Critical gap for zirconia crowns, NiTi instruments, and titanium implants where fatigue fracture is the dominant clinical failure mode (89)
15	Sensitivity Analysis	PDL modulus spans more than four orders of magnitude (0.01–175 MPa) across literature (93); systematic parametric variation rarely reported; operator meshing choices independently alter outputs	Without sensitivity analyses, differences between studies cannot be attributed to biomechanics rather than modelling decisions; model uncertainty remains unquantified (94)
16	Clinical Translation Gap	FEA predicts mechanical failure events; clinical failure involves microbiological, immunological, behavioural, and iatrogenic factors outside the model's domain	Single-template models limit generalisability to paediatric, elderly, and systemically compromised patients; mechanical adequacy does not guarantee clinical success (65, 95)

### 6.1 Fundamental limitations inherent to FEA methodology

The limitations listed in Table 3 are substantially addressable through improved *in vitro* experimental protocols, increased computational power, and enhanced material characterisation. However, a distinct and more fundamental category of limitations exists that cannot be resolved by *in vitro* approaches, arising instead from the intrinsic boundaries of FEA as a structural mechanics tool when applied to the complexity of living biological systems. These are detailed in the subsections below and represent the critical frontier for the field.

#### 6.1.1 Biological irreducibility: living tissue responses

Standard structural FEA models oral tissues as passive, inert mechanical constructs. In biological reality, alveolar bone continuously adapts its mass, architecture, and stiffness in response to mechanical stimuli through the mechanotransduction cascade described by Frost's mechanostat theory, wherein bone remodelling is regulated by strain thresholds sensed by osteocytes (58). FEA captures stress and strain at a single instant in time but cannot represent this adaptive remodelling response, meaning that long-term predictions of peri-implant bone behaviour, orthodontic bone changes, or periodontal remodelling derived from FEA alone carry inherent biological uncertainty. Similarly, vascular contributions to tissue mechanics, including pulpal hydrostatic pressure, PDL interstitial fluid dynamics, and the biphasic fluid-solid behaviour of the periodontal ligament, are not represented by structural FEA (86). The cellular and immunological responses to injury, occlusal trauma, and implant loading, which critically determine clinical healing outcomes, lie entirely outside the model's domain. This biological irreducibility is an intrinsic limitation of FEA methodology, irrespective of model fidelity.

#### 6.1.2 Implant-Bone interface assumptions

The implant-bone interface is typically modelled in dental FEA as either perfectly bonded (100% osseointegration, rigid continuity) or as a frictional contact with a defined coefficient. Neither assumption accurately represents the biological reality of osseointegration, which exists as a spatially and temporally heterogeneous continuum progressing from initial mechanical stability through primary bone deposition to mature lamellar bone contact (87). Immediately loaded implants, implants in compromised bone quality, or implants under early loading exhibit partial and regionally variable osseointegration that cannot be represented by a uniform interface assumption. This limitation is particularly consequential in studies modelling immediate loading protocols or bone quality-dependent outcomes, where the choice of interface model profoundly influences peri-implant stress predictions (43). Furthermore, the micromotion threshold beyond which osseointegration is disrupted, estimated at 50–150 micrometres in experimental models, cannot be predicted by static FEA, which does not simulate cumulative cyclic interface degradation (87).

#### 6.1.3 Muscle force modelling and masticatory loading complexity

The masticatory system generates complex, time-varying, three-dimensional force vectors through the coordinated activity of the masseter, temporalis, medial pterygoid, lateral pterygoid, and suprahyoid muscles. Van Eijden (2000) characterised the biomechanical redundancy of this system, demonstrating that jaw-closing forces cannot be uniquely determined from anatomical data alone, as multiple muscle activation patterns can produce the same resultant bite force (88). In dental FEA, masticatory loading is almost universally simplified to a single resultant force of defined magnitude applied at a fixed point and direction on the tooth or implant crown. This simplification omits the distributed, direction-dependent, and temporally dynamic nature of physiological loading, leading to systematic underestimation or mislocalisation of stress peaks, particularly in posterior segments where off-axis components are substantial (36). Occlusal contact during the chewing cycle is distributed across multiple teeth and shifts dynamically in both location and magnitude, a reality that fixed-point load models fundamentally cannot replicate. This limitation is especially consequential in TMJ, bruxism, and All-on-4 implant studies, where muscle force vectors critically determine condylar loading and prosthesis stress distribution.

#### 6.1.4 Residual stresses, thermal effects, and time-dependent material behaviour

Most dental FEA models initialise the structural domain in a stress-free reference state, ignoring the substantial residual stresses introduced during prosthetic fabrication (e.g., sintering of zirconia, pressing of lithium disilicate), cementation (polymerisation shrinkage of resin cements), and orthodontic appliance activation. These pre-existing stress states, which may approach or exceed functionally induced stresses in magnitude, directly influence fracture susceptibility and fatigue behaviour but are routinely excluded from published models. Thermal loading represents an additional underaddressed domain. Intraoral temperature fluctuations between approximately 5 degrees C and 60 degrees C during consumption of hot and cold foods generate cyclic thermal stresses in ceramic and metallic restorations that interact with mechanical stresses in fatigue accumulation. Toparli et al. (1999) demonstrated that thermal gradients in restored premolars generate stresses at the restoration-tooth interface that are of comparable magnitude to occlusal loading stresses, indicating that thermomechanical coupling is clinically non-negligible (72). Time-dependent material behaviour, including creep and stress relaxation in PMMA denture bases, silicone soft liners, and the PDL under sustained loading, is also routinely omitted, introducing systematic error in studies of removable prostheses and orthodontic appliance designs where sustained force application is central to the clinical question (86).

#### 6.1.5 Fatigue life prediction

Dental restorations, implants, and endodontic instruments are subjected to millions of fatigue loading cycles over their clinical service life. FEA provides peak stress values at a single load application, but translating these into fatigue life estimates requires additional fracture mechanics frameworks, such as S-N (stress vs. number of cycles) curves, Paris law crack propagation models, or Goodman mean-stress corrections, that most dental FEA studies do not apply (89). Without fatigue analysis, a study may report that a restoration remains below the ultimate tensile strength threshold under a single applied load while offering no information about whether cyclic stresses will accumulate sufficient damage to cause failure over clinically relevant timeframes. This gap is particularly consequential for studies of all-ceramic crowns, zirconia frameworks, NiTi rotary instruments, and titanium implant components, where fatigue fracture rather than acute overload is the dominant clinical failure mode. Incorporating fatigue life analysis through Paris law or Weibull statistical fracture approaches significantly improves the clinical predictive value of FEA models but substantially increases methodological complexity (89). Recent studies have begun to address this gap directly. Bataineh and Al Janaideh (2024) coupled FEA-derived stress fields with S-N fatigue-life prediction for CAD/CAM ceramic crowns, demonstrating that predicted survival ranged from under two years to over two decades depending on crown thickness and applied load magnitude, illustrating that static FEA peak-stress outputs alone cannot distinguish clinically viable from clinically vulnerable restoration geometries (90). A comparable fatigue-life analysis of implant-supported single crowns demonstrated that abutment material selection significantly influences predicted cycles-to-failure at the implant and abutment level even when crown material has negligible effect, a finding with direct implications for component selection in implant-supported restorations (91).

#### 6.1.6 Sensitivity analysis and operator-dependent model uncertainty

FEA results are highly sensitive to input parameter selection, yet formal sensitivity analyses examining the effect of uncertainty in material properties, boundary condition definitions, and meshing decisions on model outputs are underreported in the dental literature. Dar et al. (2002) demonstrated that statistically robust evaluation of FEA outputs requires systematic parametric variation to quantify how input uncertainty propagates to stress and displacement predictions (92). In dental applications, PDL elastic modulus, which spans more than four orders of magnitude across published studies (0.01–175 MPa) (93), represents one of the most influential and least standardised model inputs; small changes in this parameter can produce fundamentally different tooth mobility predictions and alveolar stress distributions. Similarly, the assumed degree of osseointegration at the implant interface and the assigned bone density distribution disproportionately influence peri-implant stress outputs. Beyond material property uncertainty, two researchers working from the same CBCT dataset and identical material property tables can produce meaningfully different stress results depending on their meshing strategy, boundary condition placement, and contact algorithm choices. Without sensitivity analyses, it is impossible to determine which differences between published studies reflect true biomechanical differences and which reflect operator-dependent modelling decisions (92).

#### 6.1.7 The clinical translation Gap: mechanical failure vs. clinical failure

Perhaps the most clinically significant limitation of dental FEA is the fundamental distinction between mechanical failure as predicted by the model and clinical failure as experienced by the patient. FEA identifies stress concentrations and predicts whether material strength thresholds are exceeded under defined loading conditions, but clinical outcomes are governed by a substantially broader set of determinants. Salvi and Bragger (2009) reported that peri-implant bone loss, implant fracture, prosthetic screw loosening, and veneer chipping arise from an interplay of biomechanical, microbiological, patient compliance, and iatrogenic factors that no purely mechanical model can fully account for (94). Secondary caries under composite restorations, endodontic failure due to coronal leakage, and orthodontic relapse are determined primarily by bacterial activity, coronal seal integrity, and patient compliance rather than stress magnitudes. Equally, a model predicting stress concentrations below fracture thresholds does not guarantee clinical success if parafunctional habits, bruxism, or systemic factors such as osteoporosis alter the actual loading environment from model assumptions. Population generalisability represents a further translation limitation: most published dental FEA models use a single idealised geometric template, making it difficult to extrapolate findings to paediatric patients with developing bone, elderly patients with cortical bone thinning, or systemically compromised individuals whose tissue mechanical properties differ substantially from those tabulated in standardised material property databases (13).

## 7 FEA software used in dental research

FEA in dentistry involves a pipeline of software tools spanning geometric segmentation, model assembly, numerical solving, and results visualisation. The choice of platform influences available material models, solver capabilities, and the range of analyses possible. Table 4 categorises commonly used tools by workflow phase, including dental-specific segmentation and biomechanics platforms not typically listed in general engineering references.

**Table 4:** Software used in dental FEA workflows, organised by phase.

Pre-Processing/Segmentation	Solvers	Post-Processing	Dental-Specific Tools
Hypermesh (Altair)	Nastran/OptiStruct	HyperView	Mimics/3-matic (Materialise) - CBCT segmentation
ANSA (BETA CAE)	ANSYS Mechanical	ANSYS Results	ITK-SNAP - open-source segmentation
ANSYS Workbench	Abaqus/Standard	Abaqus/Viewer	FEBio - open-source biomechanics solver
Patran (MSC)	LS-DYNA	META (BETA CAE)	MeshLab - open-source mesh processing
Abaqus/CAE	RADIOSS	LS-PrePost	Sim4Life - multiphysics biomedical platform
3-matic (Materialise)	OpenFOAM (CFD/fluid)	ParaView (open-source)	ScanIP (Simpleware) - image-based meshing

In the dental FEA literature, ANSYS Mechanical and Abaqus/Standard are the most frequently reported solver packages, due to their extensive nonlinear material libraries, validated contact mechanics algorithms, and wide documentation (95). For geometric reconstruction from CBCT data, Mimics and 3-matic (Materialise, Leuven, Belgium) are the dominant commercial platforms, enabling semi-automated segmentation of cortical bone, cancellous bone, PDL, and soft tissues. FEBio (Musculoskeletal Research Laboratories, University of Utah) is an open-source solver specifically developed for soft tissue and biomechanical applications, increasingly adopted in periodontal and PDL research due to its built-in biphasic and viscoelastic material models (96). LS-DYNA, while primarily associated with crash and impact simulation, is used in dental trauma biomechanics studies. Researchers should select software based on the specific material nonlinearities, contact conditions, and validation requirements of their study.

## 8 Advantages and limitations of FEA in dentistry

 Table 5 provides a structured summary of the principal advantages and limitations of FEA as applied to dental research.

**Table 5:** Advantages and limitations of FEA in dental research.

Advantages of FEA	Limitations of FEA
Applicable to linear, non-linear, solid, and fluid-structural interactions	Accuracy depends on quality and completeness of input data; erroneous inputs produce misleading results
Divides complex biomechanical problems into smaller, computationally manageable sub-problems	Modelling human dental anatomy is difficult due to complex geometries and incomplete knowledge of tissue mechanical behaviour
Non-invasive; allows simulation without physical specimens	Results are highly sensitive to operator assumptions and material model choice
Enables simulation of biological conditions across pre-, intra-, and post-operative stages	Validated physical properties for dental tissues (enamel, dentin, PDL, bone) remain incompletely characterised
Fully reproducible; repeated runs do not alter physical properties of the model	Simplifications (linear-elastic PDL, rigid tooth-bone interface) introduce systematic errors
Patient-specific models possible with CBCT/micro-CT imaging data	High-fidelity models carry prohibitive computational cost for routine clinical use
Cannot simulate living biological adaptation (bone remodeling, cellular mechanotransduction, immune response, wound healing)	Osseointegration interface modelled as fully bonded or frictionless; true biological continuum of osseointegration and micromotion threshold are not representable (86, 87, 96)
Masticatory muscle forces reduced to a single resultant vector; distributed, time-varying, 3D loading of the jaw system is not replicated (88)	Residual fabrication stresses, intraoral thermal cycling (5–60 degrees C), and time-dependent creep in PMMA and PDL are routinely excluded (72, 86)
Cannot predict fatigue life without S-N, Paris law, or Weibull fracture frameworks, which are absent in most dental FEA publications (89)	Sensitivity analyses underreported; PDL modulus spans more than four orders of magnitude across literature (0.01–175 MPa), making model uncertainty unquantified (92, 93)
Predicts mechanical failure, not clinical failure; bacterial, immunological, behavioural, and iatrogenic determinants of clinical outcomes are outside the model's domain (96)	Single-template geometric models limit population generalisability; findings cannot be reliably extrapolated to paediatric, elderly, or systemically compromised patients (65)

## 9 Future directions

Several technological and methodological developments hold particular promise for addressing the current limitations of dental FEA and expanding its clinical applicability.

Artificial intelligence and machine learning algorithms are being applied to automate mesh generation and material property assignment directly from computed tomography greyscale values, dramatically reducing pre-processing time and operator dependence while diminishing the subjective variability that currently limits inter-study comparability (97). Deep learning surrogate models trained on large FEA datasets can now estimate stress distributions in seconds rather than hours, opening a pathway toward routine clinical deployment (97). Real-time FEA enabled by reduced-order modelling and GPU-based computation represents an emerging area that may eventually allow intraoperative biomechanical guidance during implant placement or orthognathic surgery, with early computational frameworks already described in the surgical simulation literature (98).

These advances carry important caveats that must be stated alongside their promise. AI-assisted meshing and deep learning surrogate models are trained on existing FEA datasets; their predictive reliability degrades for anatomical geometries, loading scenarios, or material combinations underrepresented in training data, and outputs for novel clinical configurations should be interpreted with corresponding caution (97). Black-box neural network architectures generate stress estimates without mechanistic interpretability, complicating the biomechanical reasoning required for clinical translation. Crucially, AI integration does not resolve the biological irreducibility constraint identified in Section 6.1.1: no machine learning approach currently available can simulate bone remodelling, periodontal ligament fluid dynamics, or immunological responses to mechanical loading. The epistemic requirements for translating AI-augmented FEA outputs to clinical guidance therefore remain identical to those for conventional models: experimental validation under matched conditions is not rendered optional by algorithmic efficiency.

The digital twin approach involves constructing a patient-specific computational model that is continuously updated throughout the course of treatment, capturing progressive osseointegration, bone remodelling responses to orthodontic forces, and cumulative prosthetic loading history within a single evolving model (99). This represents a qualitative advance beyond the static single-load snapshots that characterise current dental FEA practice. Digital twin frameworks have already demonstrated clinical utility in cardiovascular and musculoskeletal medicine and are beginning to gain traction in dental implantology and craniofacial reconstruction (100). A dental-specific implementation of this approach has already been demonstrated: Ahn et al. (2024) coupled finite element-derived peri-implant stress data with a parametric reduced-order model to build a real-time digital twin supporting implant placement decision-making, illustrating the feasibility of translating digital twin methodology into a concrete dental clinical tool rather than a purely speculative future direction (101). Multiphysics modelling that simultaneously integrates mechanical loading, bone remodelling biology, and periodontal fluid dynamics will yield more physiologically accurate simulations and represents one of the most important near-term frontiers for the field (100). A recent comprehensive review by Falcinelli et al. (2023) identifies AI integration and multiscale modelling as the most promising near-term directions, with particular emphasis on the need for biologically informed constitutive models at the tissue scale (12).

Standardisation and reporting reform represent equally critical priorities that cannot be deferred to future computational developments. The adoption of consistent reporting protocols aligned with ASME V&V 40–2018 credibility standards (40) and the minimum reporting requirements for biomechanical FEA studies described by Erdemir et al. (2012) (41) should become mandatory expectations rather than aspirational guidelines in high-impact dental journals. A dental-specific consensus framework addressing precisely this need has now been published: the PRIFED 2026 checklist, developed through a Delphi process involving 22 international experts, retained 71 of 86 candidate reporting items spanning study design, model construction, boundary conditions, validation, and interpretation of results (102). This field-specific standard represents a substantive advance beyond the generic biomechanical frameworks previously available and gives authors, reviewers, and editors a concrete operational tool for evaluating methodological transparency in dental FEA submissions. These frameworks require authors to report mesh convergence testing, material property sources with uncertainty ranges, contact and interface model definitions, boundary condition justification, and, wherever feasible, experimental or clinical validation data. The magnitude of the current validation deficit has been directly quantified: Chang et al. (2018), reviewing 601 dental implant FEA articles published between 1997 and 2016, found that only 48 (8%) contained any form of model validation, with high-level *in vivo* validation using human subjects present in a single study (42). Enforcing these standards would substantially reduce the inter-study variability that currently prevents meaningful synthesis across the dental FEA literature. Formal sensitivity analyses examining how uncertainty in key inputs, particularly periodontal ligament elastic modulus and osseointegration interface assumptions, propagates to model outputs should similarly become a standard methodological component rather than an optional addition, as argued by Dar et al. (2002) in the context of statistical robustness in finite element modelling (92).

Rather than proposing a further bespoke reporting framework, this review recommends that authors, reviewers, and editors in dental FEA adopt the PRIFED 2026 checklist directly as the field-specific reporting standard (102), reserving local adaptation only for considerations not already addressed by its 71 retained items. Table 6 summarises the five domains of the PRIFED 2026 checklist for ease of reference during manuscript preparation and peer review.

**Table 6:** Reporting domains of the PRIFED 2026 checklist recommended for dental FEA manuscripts.

Domain	Reporting focus
Study design	Question of interest and context of use, investigated scenario, knowledge gap and clinical relevance, study objectives with varied parameters, and a statement of whether experimental validation exists (items 1a-1b, 2, 3a-3c, 4a-4e)
Model construction	Acquisition method and segmentation procedure, individual anatomical components and any omitted structures, material properties with primary references, mesh parameters (technique, element type, node and element counts), and ethical approval where clinical or animal data are used (items 5a, 5b, 5d, 5 h)
Boundary conditions	Boundary condition type and applied region, constrained degrees of freedom, load or displacement magnitude and direction, and the type of calculation and solution algorithm used (items 5c, 5e)
Validation	Verification through mesh quality control and convergence testing, and validation against experimental data or clinical outcomes where available, with justification of the comparison metrics used (item 5 g)
Interpretation of results	Comparison with relevant literature, status of the initial hypothesis, interpretation of peak stress or strain against material failure criteria, and limitations spanning model construction, material laws, boundary conditions, criteria, and validation (items 6a-6e, 7a-7e)

Finally, the field would benefit substantially from prospective interdisciplinary study designs in which FEA-derived mechanical predictions are paired with clinical or biological outcome data collected under matched conditions. The gap between mechanical failure as predicted by the model and clinical failure as experienced by the patient, identified by Salvi and Bragger (2009) as a fundamental limitation of biomechanical modelling in implantology (94), will not narrow through computational refinement alone. Closing it requires clinical researchers and computational scientists to design studies in which the model's predictions are falsifiable against real biological outcomes, establishing the kind of iterative validation loop that has driven predictive advances in cardiovascular and orthopaedic biomechanics.

## 10. Evidence mapping

Table 1 presents an evidence map summarising the methodological quality and validation status of the dental FEA literature, drawn from the best available specialty-specific systematic and scoping reviews. A consistent pattern emerges: experimental or clinical validation remains rare across every specialty for which a dedicated review exists, ranging from approximately 2.5% of implant studies to 11.6% of restorative studies, and no dedicated FEA methodological-quality review has yet been published for periodontics or for temporomandibular joint disorders and bruxism, a gap that itself reflects the relative immaturity of systematic quality appraisal in these domains (42, 47, 60, 93, 103–109).

## 11. Conclusions

Finite Element Analysis has established itself as a widely adopted computational tool in dental research, providing a non-invasive, reproducible, and quantitatively rigorous platform for biomechanical simulation across all major dental specialties.

FEA has delivered mechanistic insight into stress and strain distribution in enamel, dentin, alveolar bone, the periodontal ligament, and prosthetic materials under functional and parafunctional loading that no conventional experimental method can yield with equivalent three-dimensional resolution. Its clinical contributions span implant geometry optimisation, endodontic instrument design, prosthetic material selection, orthodontic appliance development, craniofacial trauma management, and mouthguard efficacy assessment. The most impactful advances have consistently arisen where FEA findings were validated against experimental data and subsequently translated into material guidelines, device specifications, or treatment protocols.

Nonetheless, seven inherent methodological constraints bound the clinical interpretability of these findings: the inability to represent living tissue adaptation including bone remodelling and periodontal ligament fluid dynamics; the failure of bonded or frictionless interface assumptions to capture the spatially heterogeneous osseointegration continuum; systematic mislocalisation of stress peaks when masticatory loading is reduced to a single resultant force vector; underestimation of fracture risk when fabrication residuals, thermal cycling, and time-dependent creep are excluded from models; the absence of fatigue frameworks rendering single-load peak stress values uninformative for cyclic failure prediction; unquantified model uncertainty arising from the wide literature range for periodontal ligament elastic modulus and from operator-dependent meshing decisions; and the fundamental gap between mechanical failure as predicted by the model and clinical failure as additionally governed by microbiological, behavioural, iatrogenic, and systemic determinants, which also constrains the generalisability of single-template models to paediatric, elderly, and systemically compromised populations.

These constraints collectively define the boundary conditions of FEA as an evidence-generating tool and underscore its essential role as a hypothesis-generating and design-optimisation platform rather than a definitive clinical predictor. Computational advances in artificial intelligence, GPU processing, and imaging resolution cannot resolve biology-inherent limitations. The path toward greater clinical relevance lies in mandating experimental validation, standardising reporting protocols, conducting formal sensitivity analyses as a default, and establishing interdisciplinary research programmes in which FEA findings are prospectively tested against biological and clinical outcomes. FEA is transitioning from a specialised research tool to a foundational component of evidence-based, precision dental practice, and realising that potential requires sustained collaboration between dental clinicians, biomedical engineers, and computational scientists.
